# Influence of Fibre Orientation on the Slotting Quality of CFRP Composites Using the Multi-Tooth Mill

**DOI:** 10.3390/ma17102441

**Published:** 2024-05-18

**Authors:** Ying Zhai, Shuwei Lv, Defeng Yan, Shuaishuai Wang, Junyi Lin, Chunyu Mao, Qihao Xu, Jiyu Liu

**Affiliations:** 1 School of Mechanical Engineering, Changchun Guanghua University, Changchun 130000, China; 2 School of Mechanical and Vehicle Engineering, Jilin Engineering Normal University, Changchun 130000, China; 3 School of Mechanical Engineering, Dalian University of Technology, Dalian 116000, China; yandefeng1994@163.com (D.Y.);; 4 College of Mechanical and Electrical Engineering, Northeast Forestry University, Harbin 150000, China

**Keywords:** CFRP composites, milling, fibre orientation, surface roughness

## Abstract

Carbon fibre-reinforced plastic (CFRP) composites, prized for their exceptional properties, often encounter surface quality issues during slotting due to their inherent heterogeneity. This paper tackles CFRP slotting challenges by employing multi-tooth mills in experiments with various fibre orientations and tool feed rates. In-plane scratching tests are performed under linearly varying loads; then, slotting experiments are conducted at different parameters. The scratching test results indicate that the fibre orientation and cutting angles have significant influences on forces and fracture process. The slotting experiments demonstrate that cutting forces and surface roughness *S_a_* of the bottom slotting surface are notably affected by the fibre orientation, with disparities between up-milling and down-milling sides. Reorganising *S_a_* data by local fibre cutting angle *θ* highlights consistent *S_a_* variations between up-milling and down-milling sides for 0° ≤ *θ* ≤ 90°, with lower *S_a_* on the up-milling side. However, for 90° < *θ* ≤ 150°, *S_a_* variations diverge, with lower *S_a_* on the down-milling side. Unexpectedly, *S_a_* on the down-milling side decreases with increasing *θ* in this range. Additionally, the tool feed rate exerts a more pronounced influence on *S_a_* on the up-milling side.

## 1. Introduction

Carbon fibre-reinforced plastic (CFRP) composites are ideal lightweight materials that have been widely used in aerospace, marine and automotive industries due to their high specific stiffness, excellent fatigue resistance and chemical resistance [1,2,3]. Even though various near-net-shape manufacturing technologies [4,5,6] have been developed, machining processes are still essential to obtain microstructures (e.g., edges, holes, pockets) with desired size and shape accuracy [7]. However, the heterogeneity of CFRP composites makes their machining processes more intricate compared to metal materials, resulting in serious machining damages and thus restraining their performance in practical applications [8,9,10]. Hence, it is of great significance to alleviate the machining damages and improve the surface integrity of the CFRP composites.

Previous research has reported that fibre configuration has significant influences on machining damages [11,12,13]. The fibre cutting angle *θ* with respect to edge movement is a widely used parameter that describes the cutting process of the single cutting edge on unidirectional fibres. The critical fibre cutting angle for different fibre failure modes is 90° [11], when the angle is larger than 90°, the failure modes will be further changed by increasing cutting depth [12]. An et al. [13] reported that the 150° fibre cutting angle obviously deteriorated the surface quality, resulting in severe voids, large pits and poor surface roughness. Based on the fibre cutting angle, numerous orthogonal cutting force models [14,15,16] and damage models [17,18,19] of CFRP composites were thus developed. Additionally, the milling response could be optimised by selecting suitable machining parameters or altering the fibre orientation angle *ϕ* with respect to tool feed direction [20,21,22]. However, conventional milling tools tend to result in severe surface damages, especially top-layer delamination and burrs, which cannot meet the requirement of damage-free machining. The top-layer damage occurs in the initial cutting location between the fibre and milling tool and then propagates in the critical fibre cutting angle range (0° ≤ *θ* ≤ 90°) [23,24].

To better alleviate damage, many advanced cutting tools have been designed, such as left–right edge mills [25], nicked edge mills [26,27], micro-textured mills [28], various multi-tooth mills [29,30], and integrated-micro hybrid mills [31]. Multi-tooth mills, in particular, have proven to be effective and economical for slotting or routing CFRP composites, which can achieve minimal cutting depth per tooth to reduce damage [32]. Their left–right edge structure effectively suppresses the top-layer delamination and burrs [25,33]. The excellent side milling quality obtained by multi-tooth mills has attracted considerable research attention [26,29,34]. However, the influence of fibre cutting angles on the bottom slotting surfaces has been rarely reported, especially the insufficient investigation of changes in milling direction, which has been focused on in previous studies of damage behaviours on the side milling surface [34,35,36]. Moreover, since the material removal process generated by the micro-cutting edges of a multi-tooth mill is closely related to a taper grooving process by a sharp indenter, scratching tests of CFRP under different fibre orientations is crucial for optimising parameters for low-damage milling using multi-tooth mills. As the chip thickness and fibre cutting angle during the actual milling process both vary continuously, while previous studies have provided insights into the scratching process with typical fibre cutting angles (0°/45°/90°/135°) [37,38,39], scratching tests under subdivided fibre cutting angles with continuous loading variation are necessary to better understand damage behaviours on the bottom slotting surface.

Therefore, in this paper, we aim to analyse the effects of the fibre orientation on the bottom slotting performance of multi-tooth mills by conducting in-plane scratching tests of unidirectional CFRP composites and slotting experiments with different fibre orientation angles. Through comparison of scratching damage behaviours and bottom slotting surface quality under various fibre orientation angles, we seek to elucidate how fibre orientation influences the performance of multi-tooth mills in bottom slotting applications.

## 2. Experimental Details

### 2.1. Setup of Scratching Experiment

**Figure 1:**
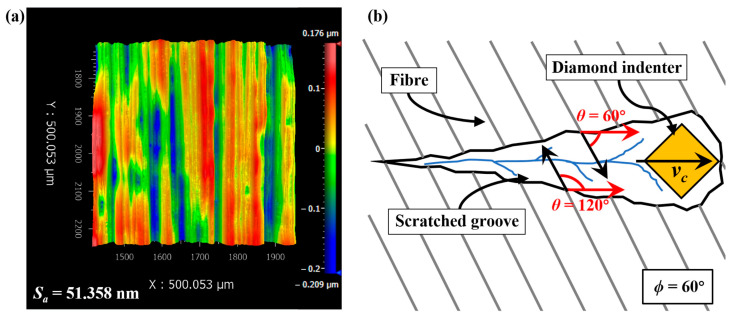
Detailed explanation of the scratching tests: (**a**) surface profile of the polished specimens for scratching, and (**b**) a schematic diagram of the scratching process when *ϕ* = 60°.

The workpiece materials were unidirectional CFRP composites, with T300-3K carbon fibres as the reinforcement. Mechanical properties of the 7 μm diameter T300 fibres from Toray are outlined as follows: 230 GPa axial tensile modulus, 27 GPa axial shear modulus, 15 GPa radial tensile modulus, 7 GPa radial shear modulus, and 0.013 axial Poisson’s ratio of fibre [40]. Before conducting the scratching tests, 20 mm × 10 mm × 3 mm blocks were cut from the unidirectional CFRP composites and mechanically polished to minimise the influence of the original surface texture. The surfaces were sequentially polished with 400#, 800#, 1200# sandpapers and w7, w3.5, w1.5, and w0.5 diamond slurry. After careful polishing, surface profile of the specimens was inspected by a 3D surface optical profilometer (NewView-9000, Zygo Co., Ltd., Middlefield, CT, USA), as shown in Figure 1a. The surface roughness *S_a_* was less than 100 nm, which could meet the requirements for scratching tests. The scratching tests were conducted on a scratch tester (Micro/nano-Scratcher-1000-V, Huang Lab, Jilin University, Changchun, China) using a standard Vickers diamond indenter [41]. The Vickers diamond indenter had pyramid shape, and the edge was consistently maintained facing forward during the scratching tests. In each scratching trial, the indenter load linearly increased from 0 mN to 1000 mN, with a scratching length of 1000 μm and a scratching speed of 10 μm/s. The variations in normal force and tangential force with displacement were recorded. To elucidate the effect of fibre cutting angles on failure modes, four typical values of fibre orientation angle *ϕ*: 0°, 30°, 60°, and 90° were adopted. As shown in Figure 1b, the two sides of scratched groove indeed exhibited two different fibre cutting angles under one fibre orientation angle, and thus 0°, 30°, 60°, 90°, 120° and 150° of the fibre cutting angles *θ* were involved. To reveal the material removal behaviour and damage characteristics during the CFRP scratching process, a super high magnification lens zoom 3D microscope (VHX-600E, Keyence Co., Ltd., Osaka, Japan) and a scanning electron microscope (SEM) (QUANTA 450, FEI Co., Ltd., Hillsboro, TX, USA) were employed to observe the scratched grooves [42].

### 2.2. Setup of Slotting Experiment

**Figure 2:**
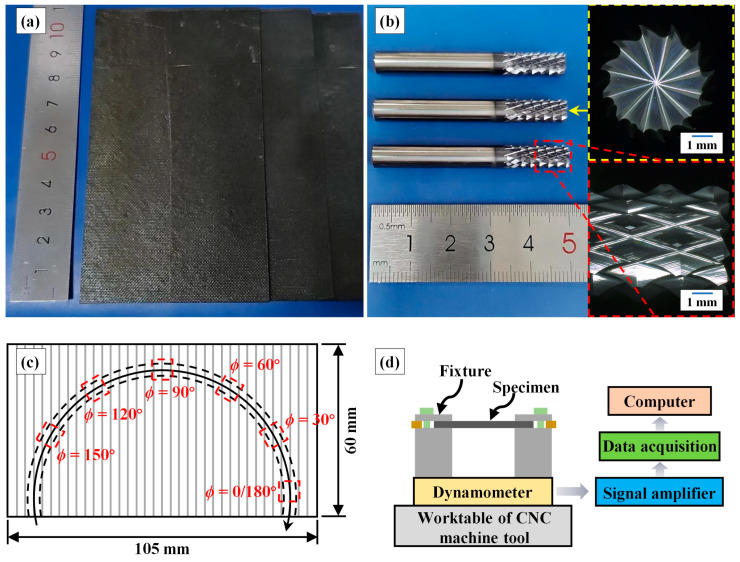
Detailed explanation of the slotting experiments: (**a**) slotting specimens, (**b**) milling tools, (**c**) schematic diagram of the tool path, and (**d**) experimental setup.

The slotting specimens were the same unidirectional CFRP composites used for the scratching tests, divided into 105 mm × 60 mm × 3 mm plates, as shown in Figure 2a. The slotting experiments employed 6 mm diameter carbide multi-tooth mills with diamond coating, as shown in Figure 2b. During the slotting experiments, the milling tool moved along an arcuate trajectory with a diameter of 80 mm, gradually altering the tool feed direction to achieve machining with continuously varying fibre orientation angle *ϕ*, as illustrated in Figure 2c. The cutting parameters referred to some previous experimental works [34,43]. We investigated the slotting quality with different cutting depths per tooth by utilising the tool feed rate as a variable. The machining parameters for the slotting experiments are listed in Table 1. All experimental trails were conducted at an ambient temperature of 20 °C, and new milling tools were used for each trail to eliminate the effect of tool wear. Dry milling was adopted to avoid coolant affecting CFRP machinability [44,45]. Each slotting trail was repeated at least three times. As shown in Figure 2d, the cutting forces were measured using a three-directional dynamometer (9257B, KISTLER Co., Ltd., Winterthur, Switzerland); after signal amplifier and data acquisition, the force data were transmitted to a computer for recording. After slotting, to distinguish different milling directions (up-milling and down-milling), two measurement zones were selected on each position with selected *ϕ*. The two measurement zones were symmetrically distributed on the bottom surface of the slot with a spacing of 3 mm. The NewView-9000 profiler was employed to measure the milling surface morphology, with a measurement area of 400 μm × 400 μm, which was determined based on previous works [46,47]. Due to its ability to provide more comprehensive surface information, the arithmetic mean deviation *S_a_* derived from the measurement results was used to evaluate the surface quality [36].

## 3. Results and Discussion

### 3.1. Detailed Scratching Results

**Figure 3:**
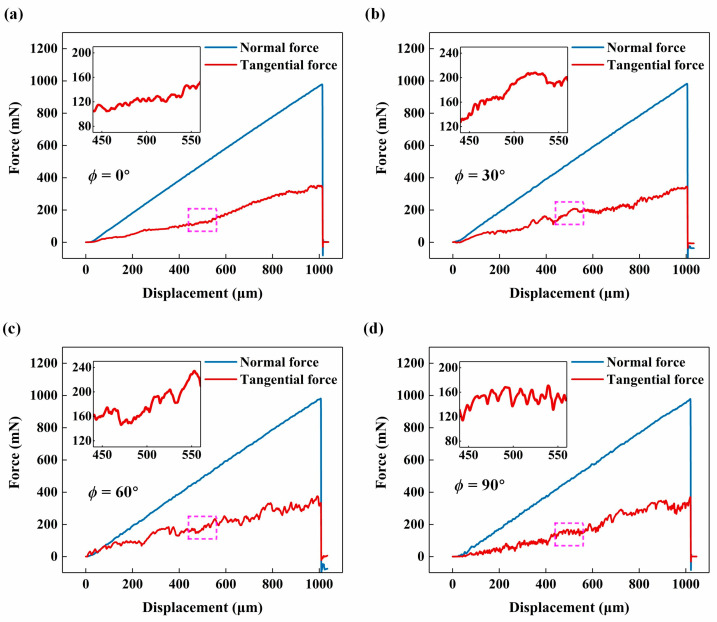
Force variations with displacement during scratching tests under different fibre orientation angles *ϕ*: (**a**) *ϕ* = 0°, (**b**) *ϕ* = 30°, (**c**) *ϕ* = 60°, and (**d**) *ϕ* = 90°.

Figure 3 shows the variations in normal and tangential forces during the scratching tests. The tangential force exhibited a similar linear increase as the applied normal force linearly increased from 0 mN to 1000 mN as described in Section 2.1 but showed more pronounced fluctuations. The tangential forces in the same displacement range (440 μm to 560 μm) were magnified to better observe their differences. When the fibre orientation angle *ϕ* was 0°, the fluctuations in tangential force were relatively lower, as shown in Figure 3a. As *ϕ* increased, the fluctuations in tangential force became more obvious, as shown in Figure 3b–d. When *ϕ* = 90°, the tangential force displayed frequent and typical periodic fluctuations, with an amplitude of approximately 20 mN (Figure 3d), indicating periodic removal of fibre and matrix. By contrast, when *ϕ* = 30° or *ϕ* = 60°, although the fluctuations in tangential force were less frequent, the amplitudes were much larger (Figure 3b,c), suggesting that these values of *ϕ* might contribute to sudden and significant fibre fractures. The ratio of tangential force to normal force increased from 28% (*ϕ* = 0°) to 41% (*ϕ* = 30°), then further increased to 43% (*ϕ* = 60°), and finally decreased to 36% (*ϕ* = 90°). This demonstrated that the scratching process under *ϕ* = 30° (combination of two fibre-cutting cases at *θ* = 30° and *θ* = 150°) or *ϕ* = 60° (combination of two fibre-cutting cases at *θ* = 60° and *θ* = 120°) would result in more severe fibre fractures and other damages.

**Figure 4:**
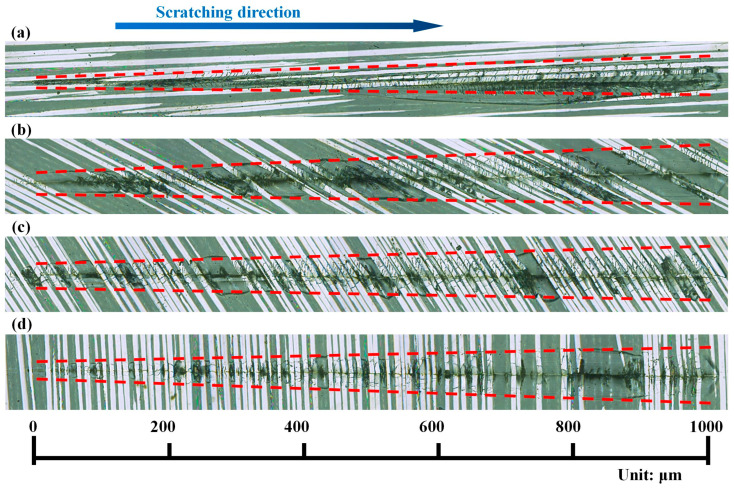
Optical images of the scratched grooves (highlighted with red dashed lines) obtained at different fibre orientation angles *ϕ*: (**a**) *ϕ* = 0°, (**b**) *ϕ* = 30°, (**c**) *ϕ* = 60°, and (**d**) *ϕ* = 90°.

**Figure 5:**
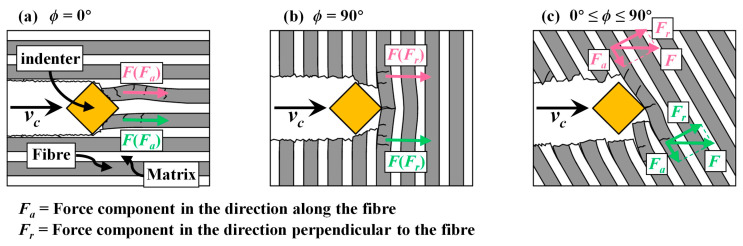
Schematics of scratching damage behaviours at different fibre orientation angles *ϕ*: (**a**) *ϕ* = 0°, (**b**) *ϕ* = 90°, and (**c**) 0° ≤ *ϕ* ≤ 90°.

**Figure 6:**
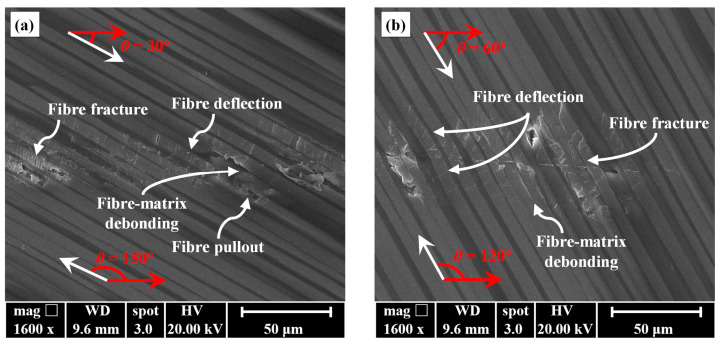
SEM images of the scratched grooves obtained at different fibre orientation angles *ϕ*: (**a**) *ϕ* = 30°, and (**b**) *ϕ* = 60°.

Optical images of scratched grooves with varying *ϕ* measured by the super high magnification lens zoom 3D microscope VHX-600E are shown in Figure 4. Figure 5 presents schematics of scratching damage behaviours at different *ϕ*. As can be seen in Figure 4a, when *ϕ* = 0°, the fibres in the scratching area were mostly crushed, but the radial damage size perpendicular to the scratching direction was minimal, resulting in a narrower scratched groove. This is because the fibre was primarily subjected to axial compression, causing it to buckle and ultimately resulting in fibre fracture, as shown in Figure 5a. As *ϕ* increased, the length of crushed fibres decreased rapidly, and the radial damage size significantly increased, as shown in Figure 4b–d. However, the growth of the radial damage size almost stopped once the fibre orientation angle *ϕ* reached 30°. This might be attributed to the fact that the radial damage size was influenced by both sin*ϕ* and the fibre fracture length, which would be quantified later. The radial damage sizes at *ϕ* = 30° and *ϕ* = 60° were similar, while that at *ϕ* = 90° became smaller. The scratching damage behaviour at *ϕ* = 90° is illustrated in Figure 5b. The indenter radially pushed the fibre, causing the fibre to experience a first shear fracture. As the scratching process continued, cracks propagated at the fibre–matrix interface, and the fibre underwent greater fibre deflection. Eventually, when the bending load exceeded the bending strength of the fibre, a bending fracture occurred. Moreover, apart from symmetric scratching instances at *ϕ* = 0° and *ϕ* = 90°, the radial damage sizes on two sides of the scratched groove exhibited non-uniformity due to varying values of fibre cutting angle *θ* on each side, as shown in Figure 5c. SEM images magnified at 1600× revealed fibre deflection, fracture, fibre–matrix interface debonding, and even fibre pullout, as shown in Figure 6. Remarkably, fibre–matrix interface debonding and fibre pullout predominantly occurred on the side where *θ* ≥ 90°, consistent with findings reported by Shi et al. [38]. This disparity in damage distribution stems from distinct fibre failure modes: tensile-induced fracture on the side where *θ* ≤ 90°, and compression-induced bending leading to fracture on the side where *θ* ≥ 90°. This observation suggests that the fibre orientation and cutting orientation have obvious influences on the failure mechanisms, shedding light on the complex damage behaviours during CFRP slotting processes.

**Figure 7:**
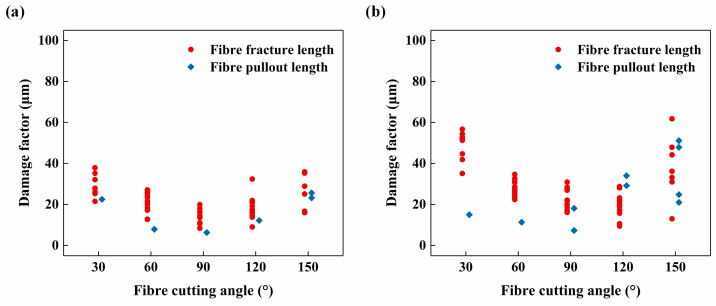
Fibre fracture and fibre pullout lengths at different fibre cutting angles *θ*: (**a**) the results measured in the displacement range from 200 μm to 400 μm, and (**b**) the results measured in the displacement range from 600 μm to 800 μm.

The length-based damage factors have been widely adopted in previous references [32,48]. Using two length-based damage factors (fibre fracture length and fibre pullout length, which were directly measured along the fibre direction from the centreline of the scratched groove), the scratching damages were quantitatively evaluated in two displacement ranges (200 μm to 400 μm and 600 μm to 800 μm, respectively). Based on the fibre cutting angles *θ* on both sides of the scratched grooves, the damage results at different fibre cutting angles *θ* were directly measured, as shown in Figure 7a,b. As *θ* increased, both the fibre fracture length and fibre pullout length decreased in the range from *θ* = 30° to *θ* = 90° and then increased. By comparing Figure 7a,b, doubling the applied normal force resulted in a 50% increase in the damage factors, indicating that reducing cutting force was indeed beneficial for suppressing damage. It could be also observed that obtuse fibre cutting angles resulted in more fibre pullouts. Referring back to Figure 4, it is evident that acute fibre cutting angles result in more fibre fractures but fewer fibre pullouts and fibre–matrix interface debonding compared to obtuse fibre cutting angles. Specifically, the fibre fractures caused by acute fibre cutting angles take the form of micro-cracks at an about 45° inclination to the scratch direction due to cone crack propagation, while the fibre fractures caused by obtuse fibre cutting angles perpendicularly penetrate the entire fibre due to fibre bending. After summarising and averaging all the damage factor data from Figure 7 according to the fibre orientation angle *ϕ*, the radial damage size of scratched grooves at different *ϕ* can be calculated. There was a nonlinear relationship between the radial damage size and the value of *ϕ*. The calculation results indicated that the radial damage size was minimal at *ϕ* = 0°, then rapidly increased with the increment of *ϕ* from 0° to 30°, followed by a slight increase as *ϕ* increased from 30° to 60°, and finally slightly decreased at *ϕ* = 90°, as mentioned earlier. This is because as *ϕ* increased, the fibre fracture length gradually decreased, while its proportion projected onto the radial direction of the scratched groove gradually increased according to sin*ϕ*. Within 30° ≤ *ϕ* ≤ 60°, the decrease and increase rates have basically reached equilibrium. Therefore, the fibre orientation angle had obvious influences on the damage behaviours of the CFRP. The *ϕ* = 0° (fibre cutting cases at *θ* = 0°/180°) and *ϕ* = 90° (fibre cutting cases at *θ* = 90°) were favourable for alleviating fibre fracture and pullout.

### 3.2. Slotted Surface Quality Comparison

**Figure 8:**
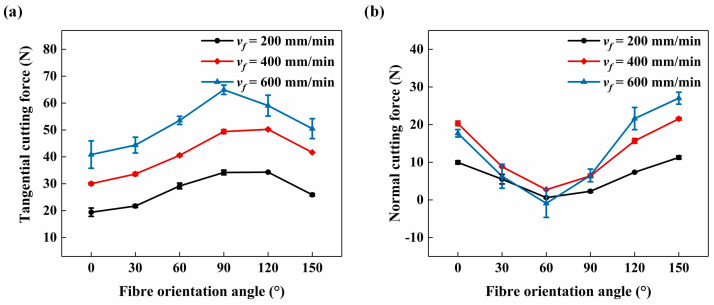
Cutting forces with increasing *ϕ*: (**a**) tangential cutting force, and (**b**) normal cutting force.

**Figure 9:**
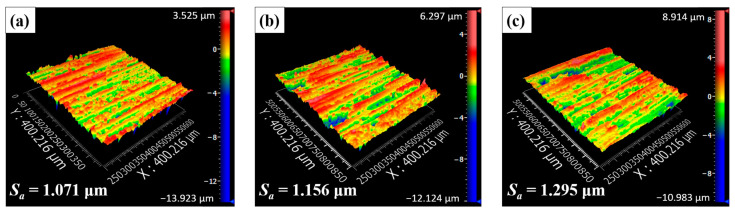
Surface morphology measured on the up-milling side when *ϕ* = 90°: (**a**) *v_f_* = 200 mm/min, (**b**) *v_f_* = 400 mm/min, and (**c**) *v_f_* = 600 mm/min.

**Figure 10:**
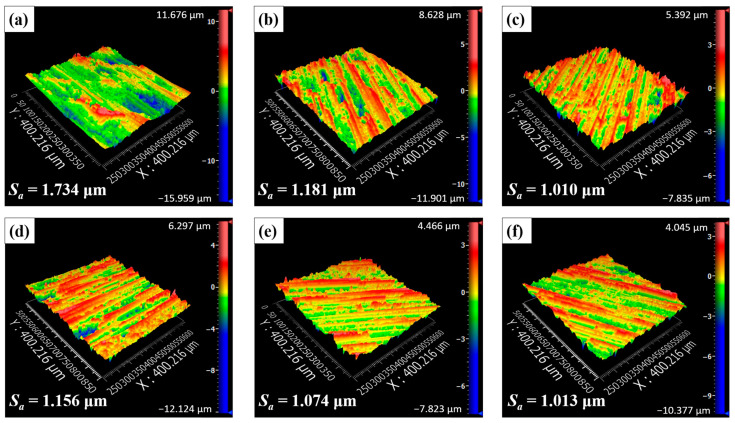
Surface morphology measured on the up-milling side when *v_f_* = 400 mm/min: (**a**) *ϕ* = 0°, (**b**) *ϕ* = 30°, (**c**) *ϕ* = 60°, (**d**) *ϕ* = 90°, (**e**) *ϕ* = 120°, and (**f**) *ϕ* = 150°.

Prior to comparing the slotting surface quality, cutting forces at different fibre orientation angles are shown in Figure 8. It was evident that the tool feed rate had a significant impact on the cutting forces, with the tangential cutting force being affected more obviously. Figure 9 presents surface morphology images when *ϕ* = 90° with varying tool feed rates on the up-milling side of the bottom slotting surface. These images revealed that higher tool feed rates deteriorated CFRP surface integrity, resulting in more serious fibre fractures and debonded grooves. As the tool feed rate increased from 200 mm/min to 600 mm/min, the surface roughness *S_a_* increased by approximately 20%. Furthermore, Figure 8 demonstrates that the cutting forces are also significantly influenced by the fibre orientation angle *ϕ*. As *ϕ* increased, the tangential cutting force increased with *ϕ* varying from 0° to 90° and then decreased. Conversely, the normal cutting force decreased with *ϕ* increased from 0° to 60°, and then rapidly increased. Figure 10 shows surface morphology measured at different *ϕ* on the up-milling side of the bottom slotting surface. The surface morphology when *ϕ* = 0° was the most irregular, with significant differences from other cases.

**Figure 11:**
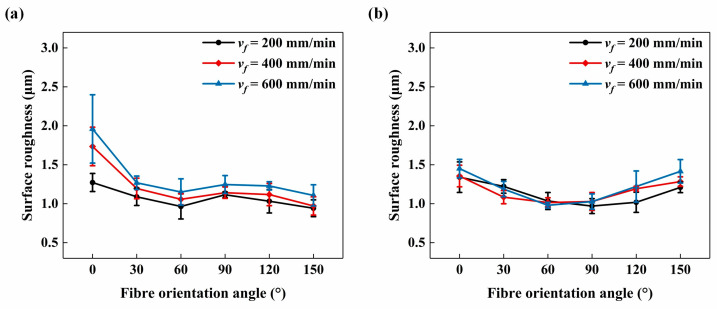
Surface roughness with increasing *ϕ*: (**a**) the results measured on up-milling side, and (**b**) the results measured on down-milling side.

The changes in surface roughness *S_a_* measured at different *ϕ* are illustrated in Figure 11. Figure 11a presents results from the measurement zone on the up-milling side, while Figure 11b displays results from the measurement zone on the down-milling side. Notably, increasing the tool feed rate led to a noticeable increase in *S_a_* on the up-milling side, whereas its influence on the down-milling side was less pronounced. Moreover, at each milling direction, *S_a_* was significantly influenced by *ϕ*, albeit with variations observed between the up-milling and down-milling sides. Specifically, on the up-milling side, *S_a_* was markedly impacted by *ϕ*. As *ϕ* increased, *S_a_* gradually decreased within the range from *ϕ* = 0° to *ϕ* = 60°, followed by an increase at *ϕ* = 90° and a subsequent decrease. On the down-milling side, *S_a_* exhibited a gradual decrease with increasing *ϕ*, reaching its minimum at *ϕ* = 60° or *ϕ* = 90°, before gradually increasing again. By varying *ϕ*, the improvement of *S_a_* on the bottom slotting surface was approximately 43%, which was lower than that on the side milling surface (approximately 72% [40]). Even though there were some similarities in the *S_a_* variations caused by different *ϕ* in the up-milling and down-milling sides, the underlying influence mechanisms behind the similarities, considering the differences in *θ* at the measurement zones, need to be further analysed in Section 3.3.

### 3.3. Influence of Fibre Cutting Angles on the Slotting Process

**Figure 12:**
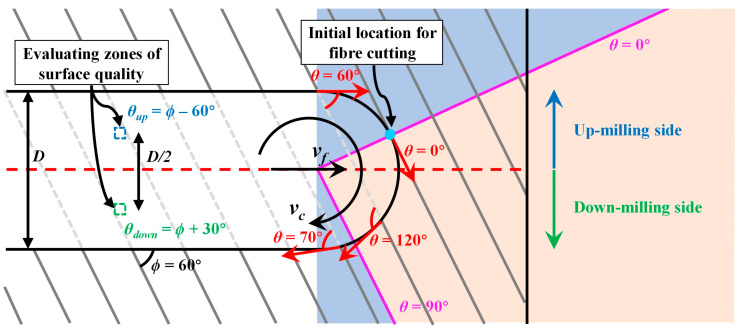
Schematic diagram of the *θ* variation during slotting CFRP when *ϕ* = 60°.

To better analyse the influence of fibre cutting angle *θ* on the slotting process, surface roughness data were rearranged based on the representative *θ* values at the 400 μm × 400 μm measurement zones (*θ_up_* and *θ_down_*). Figure 12 presents a detailed schematic diagram of the *θ* variation during the slotting process. Regardless of *ϕ*, *θ* values spanned the entire range from 0° to 180° due to the constant engagement of the mill, forming a semi-cylindrical contact area with the CFRP plates. However, considering the variations in cutting depth per tooth at different evaluating positions due to the rotational motion and the feed motion of the mill, as well as the differences in damage propagation contributed by varying initial locations for fibre cutting, the distribution of *θ* along the macro contact area necessarily resulted in different milling responses. Taking the slotting process at *ϕ* = 60° as an example, the initial location for fibre cutting occurred on the up-milling side of the bottom slotting surface. After the fibre was cut off, the fibre ends on both up-milling and down-milling sides underwent multiple-pass cutting with varying *θ* [49], ranging from 0° to 60°, and 180° (0°) to 60°, respectively. This fact influenced the propagation of fibre damage, which had been reported in previous studies of top-layer damages [23,24]. Even in homogeneous materials, multiple-pass cutting could also complicate the damage behaviours [50,51]. As for CFRP composites, the multiple-pass cutting will further result in a significant uncut fibre deflection and continuous propagation of fibre–matrix interface debonding. Such unique damage behaviours during the milling process could not be observed in single-path scratching or orthogonal cutting.

**Figure 13:**
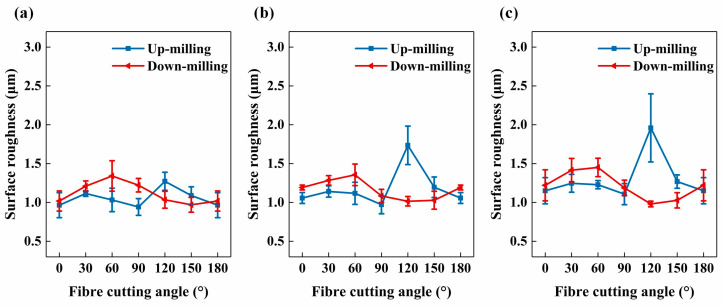
Surface roughness with increasing *θ* for both up-milling and down-milling sides: (**a**) *v_f_* = 200 mm/min, (**b**) *v_f_* = 400 mm/min, and (**c**) *v_f_* = 600 mm/min.

Table 2 shows the distribution of *θ* on the up-milling and down-milling sides during the slotting process at different *ϕ*, as well as the corresponding fibre cutting angles *θ_up_* and *θ_down_*. Figure 13 illustrates the variation of *S_a_* with increasing *θ* on both up-milling and down-milling sides, which was reorganised based on the data from Figure 11 according to Table 2. When 0° ≤ *θ* ≤ 90°, *S_a_* was lower on the up-milling side than the down-milling side, but the variation trends of *S_a_* were generally consistent. The *S_a_* increased with *θ* increased from 0° to 60°, and then gradually decreased. However, when 90° < *θ* ≤ 150°, the *S_a_* on the up-milling side increased again with *θ* increased from 90° to 120° and then decreased. In contrast, on the down-milling side, the *S_a_* continued to decrease with *θ* increased from 90° to 120° or 150° before increasing again. Therefore, when 90° < *θ* ≤ 150°, *S_a_* was lower on the down-milling side than on the up-milling side. In summary, the variation in the *S_a_* on the up-milling side of the bottom slotting surface was basically consistent with the abovementioned scratching results and the previous orthogonal cutting results from Xu et al. [19]. However, in the case of down-milling, there was an unforeseen reduction in *S_a_* as *θ* increased when 90° < *θ* ≤ 150°. This phenomenon could be attributed to the gradual reduction in cutting depth per tooth during the down-milling process, ranging from its maximum value to zero. This gradual reduction of cutting depth per tooth may have induced intricate fibre deflection and failure modes, which could not be observed in conventional scratching or orthogonal cutting tests where the cutting depth either increased or remained constant. Similar significant differences of milling response caused by different milling directions have also been reported in previous study focusing on the top-layer damage conducted by Wang et al. [52], which indicates that down-milling direction indeed has a significant impact on fibre damages.

## 4. Conclusions

In this paper, we investigated the effects of fibre orientation angle *ϕ* on cutting forces and slotting quality, while also analysing the varying fibre cutting angle *θ* during the slotting process. The influences of fibre cutting angle *θ* on damage mechanisms and surface roughness *S_a_* under different milling directions were also studied. The conclusions could be summarised as follows:

1. During scratching tests, as *ϕ* increased, the length of fractured fibre and the number of fractures decreased, but the fractures became gradually more severe.
2. In asymmetric scratching cases, it was observed that the side with a *θ* greater than 90° experienced significantly more damage than that with a *θ* less than 90°, especially fibre–matrix interface debonding and fibre pullouts.
3. The cutting forces during the slotting process were significantly influenced by both *ϕ* and tool feed rate, indicating that the mechanical behaviour during slotting could be effectively adjusted by the *ϕ*.
4. The *S_a_* on the bottom surface of the CFRP slot was also influenced by the *ϕ*. The tool feed rate increased the *S_a_* on the up-milling side but had a less obvious effect on the down-milling side.
5. After reorganising the *S_a_* data based on the *θ* in the measurement zone, it was evident that the variations of *S_a_* for both up-milling and down-milling sides were consistent when 0° ≤ *θ* ≤ 90°, but different when 90° < *θ* ≤ 150°.

In summary, this study systematically analysed the effect of fibre orientation on damage behaviour and surface quality during the slotting process, which might provide valuable insights for a deeper understanding of the slotting mechanisms of CFRP composites and optimising slotting processes.

## Figures and Tables

**Table 1:** Machining parameters of the slotting experiments.

Machining Parameters	Values
Cutting speed *v_c_*	120 m/min (6370 r/min)
Axial infeed *a_p_*	1.5 mm
Feed rate *v_f_*	200, 400, 600 mm/min
Fibre orientation angle *ϕ*	0°, 30°, 60°, 90°, 120°, 150°

**Table 2:** Variations in fibre cutting angles at different fibre orientation angles.

*ϕ*	Up-Milling Side		Down-Milling Side	
	Variation in *θ*	*θ_up_*	Variation in *θ*	*θ_down_*
0°	[180°, 90°]	120°	[90°, 0°]	60°
30°	[30°, 0°]∪[180°, 120°]	150°	[120°, 30°]	90°
60°	[60°, 0°]∪[180°, 150°]	180/0°	[150°, 60°]	120°
90°	[90°, 0°]	30°	[180°, 90°]	150°
120°	[120°, 30°]	60°	[30°, 0°]∪[180°, 120°]	180/0°
150°	[150°, 60°]	90°	[60°, 0°]∪[180°, 150°]	30°

## Data Availability

The original contributions presented in the study are included in the article, further inquiries can be directed to the corresponding authors.
